# Generative Adversarial Network-Based Fault Detection in Semiconductor Equipment with Class-Imbalanced Data

**DOI:** 10.3390/s23041889

**Published:** 2023-02-08

**Authors:** Jeong Eun Choi, Da Hoon Seol, Chan Young Kim, Sang Jeen Hong

**Affiliations:** Department of Electronics Engineering, Myongji University, 116 Myongji-ro, Yongin-si 17058, Gyeonggi-do, Republic of Korea

**Keywords:** fault detection, generative adversarial networks, machine learning, optical emission spectroscopy, plasma etch

## Abstract

This research proposes an application of generative adversarial networks (GANs) to solve the class imbalance problem in the fault detection and classification study of a plasma etching process. Small changes in the equipment part condition of the plasma equipment may cause an equipment fault, resulting in a process anomaly. Thus, fault detection in the semiconductor process is essential for success in advanced process control. Two datasets that assume faults of the mass flow controller (MFC) in equipment components were acquired using optical emission spectroscopy (OES) in the plasma etching process of a silicon trench: The abnormal process changed by the MFC is assumed to be faults, and the minority class of Case 1 is the normal class, and that of Case 2 is the abnormal class. In each case, additional minority class data were generated using GANs to compensate for the degradation of model training due to class-imbalanced data. Comparisons of five existing fault detection algorithms with the augmented datasets showed improved modeling performances. Generating a dataset for the minority group using GANs is beneficial for class imbalance problems of OES datasets in fault detection for the semiconductor plasma equipment.

## 1. Introduction

Miniaturization of the semiconductor device technology has incorporated a more stringent process control in the plasma process than ever before. Changes in the condition of components of semiconductor equipment can affect the plasma inside the process chamber, which in turn affects process results. [1,2]. Advanced process control (APC) is recommended to detect small changes in equipment parts and accurately detect equipment faults to prevent an expected misprocessing. Previously, statistical process control was used to detect and control faults using statistical methods [3,4]. However, fault detection using conventional statistical methods became challenging due to increased off-line post-processing metrology. Thus, APC was suggested to improve the control issues that occur in the semiconductor manufacturing process [5].

Attempts have been made to detect faults in semiconductor processes, and accurate fault detection leads to positive results in the process yield and throughput [6,7]. The plasma etching process that requires more stringent process controls held a limit to detecting faults from equipment state variable identification (SVID) data, and the in-situ sensors, such as optical emission spectroscopy (OES) and plasma impedance monitor, were employed to enhance the scientific evidence for anomalies based on plasma-related phenomenology [8,9,10]. OES is a non-invasive plasma monitoring sensor that measures chemical species in plasma using the spectroscopic phenomena of gas-phase atomic/molecular species [11]. It is commonly used for detecting the etching process endpoint and has been actively investigated for fault detection of plasma equipment using statistics-based modeling approaches [12,13]. A high-performance model can be implemented by manipulating OES data to obtain plasma information, including electron temperature and electron density [14,15], or by selecting radical peaks related to the process based on domain knowledge [16].

Machine learning algorithms have been applied to improve the fault detection performance in complex plasma processes using OES data [17,18]. Data in the semiconductor mass production environment, including OES data, are rarely labeled and contain extremely small fault data compared with the data from the normal process. They also suffer from data class imbalances [19,20]. Class imbalance causes the problem of insufficiently predicting a minority class by intensively learning a majority class in machine learning, and failure to classify a faulty wafer, which is mostly the minority class, leads to large production loss in wafer fabrication [21,22].

The class imbalance problem in the data modeling of fault detection has been treated as an important issue in various fields, including the semiconductor industry. To mitigate the class imbalance problem in data modeling, strong algorithms against the class imbalance or methods of generating additional artificial data have been applied [22,23,24]. Fault detection using one-class classification models has been researched in the current semiconductor process diagnosis to address data imbalance [25]. For more delicate APC in the semiconductor process, it is becoming more important not only to detect process faults, but also to identify the cause of the faults, but one-class classification algorithms have limitations in classifying the cause. One of the methods used at the data-level, the undersampling method, is largely not used because it removes potentially valuable information, leading to a loss of data and changing the overall distribution of the data [26]. Moreover, the random over-sampling method is preferred over the random under-sampling method because there is no loss of minor class data. However, since it is randomly replicated, it may be similar to the repetition of an actual sample, which does not bring about data diversity and may cause overfitting [27]. In general, the synthetic minority oversampling technique (SMOTE) or adaptive synthetic algorithm, which are the most used, generate new samples based on neighbors, but since they do not consider the entire data of the minority class, there is a possibility of generating biased artificial data [28]. In addition, over-sampling methods, such as SMOTE cannot reduce classification bias towards the majority class in high-dimensional imbalanced data, and the Euclidean distance used in SMOTE is not an appropriate index to measure the similarity of samples in a high-dimensional space [29]. To overcome the limitations of these algorithms, extended addressing imbalanced data studies and methods applying generative adversarial networks (GANs) have been studied [30]. Unlike the SMOTE method, which does not reflect the data distribution, the data generated using GANs capture the distribution of the trained data. In addition, since a GAN is based on the neural networks, it is useful to increase performance compared to SMOTE, which has poor control flexibility, because it can control a specific field by tuning many parameters [31]. It has the advantage that high-quality artificial data can be generated even with very small samples [32]. In view of the characteristics of the GAN mentioned above, it is determined that the GAN is appropriate as a tool used to balance data in a semiconductor domain where high-dimensional data is acquired and the degree of class imbalance is very severe.

**Figure 1:**
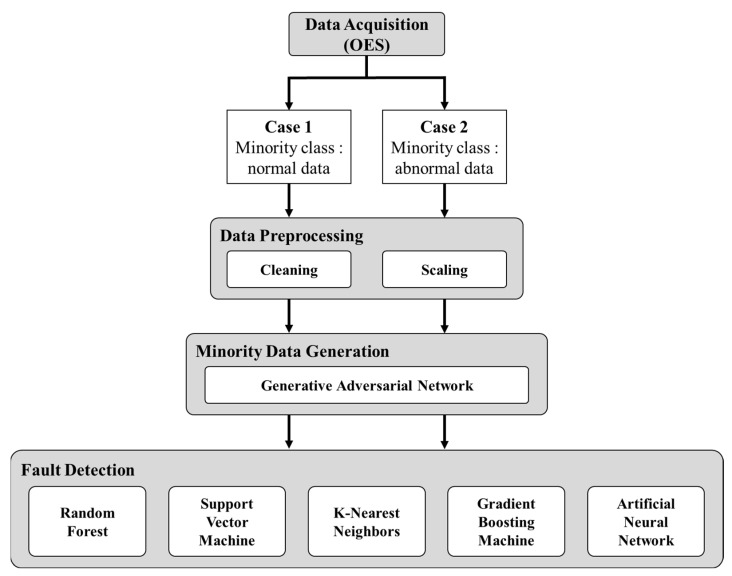
Flowchart of the fault detection using the GAN.

To reduce wafer scrap and improve yield in the semiconductor industry, resolving the class imbalance of data for fault detection is essential. Although using a plasma monitoring sensor for fine control in the plasma etching process is necessary, studies on the class imbalance problem for plasma measuring sensor data have hardly been conducted. Previously, we conducted studies on the fault detection of plasma equipment using OES, a non-invasive plasma measurement sensor with a high usability, and the models suffered from a class imbalance problem in the modeling work [33]. OES data, which suffer from a serious class imbalance in actual mass production, are high dimensional plasma measurement data widely used for APC, and they are required to solve the class imbalance problem of OES data. In this paper, we propose a fault detection method for alleviating the class imbalance phenomenon of OES data collected from plasma etching equipment using GANs, as shown in Figure 1. Here, we construct a model that detects a fault in the plasma etching equipment part level, and we acquired the scenario-based two datasets of the normal and abnormal states of a mass flow controller (MFC) for SF_6_ in the silicon trench etching process using SF_6_, O_2_, and Ar gas mixture Following the application of the GAN to both datasets, the improvement results of the fault detection algorithm are analyzed.

## 2. Data Acquisition and Preprocessing

The employed etching equipment for the OES data acquisition was a 300 mm Kiyo 45 etch, manufactured by LAM Research, which has a transformer-coupled plasma-reactive ion etching with a 13.56 MHz radio frequency (RF) generator. To acquire real-time plasma data during the etching process, the OES was mounted on the outside chamber via a viewport. The target process was a silicon trench etch through plasma injected with SF_6_, O_2_, and Ar gas, and 20 × 20 mm^2^ sized silicon pattern samples with a SiO_2_ etch mask grown on 300 mm silicon wafer, supplied by Samsung Electronics through the pattern wafer program via the Korea Semiconductor Industry Association, were used. In the pattern wafer, the thickness of the SiO_2_ etch mask was 2400 nm, and the bar type trench etch with a 200 nm trench width was targeted. Patterned samples after the etching process were measured using the scanning electron microscope (SEM) with a 3.5 nm resolution to obtain etch results, including etching geometries of the profile and depth. The real-time data of the equipment, including OES data, were specified as SVID and stored in a database (DB) using high-speed SECS message services.

**Figure 2:**
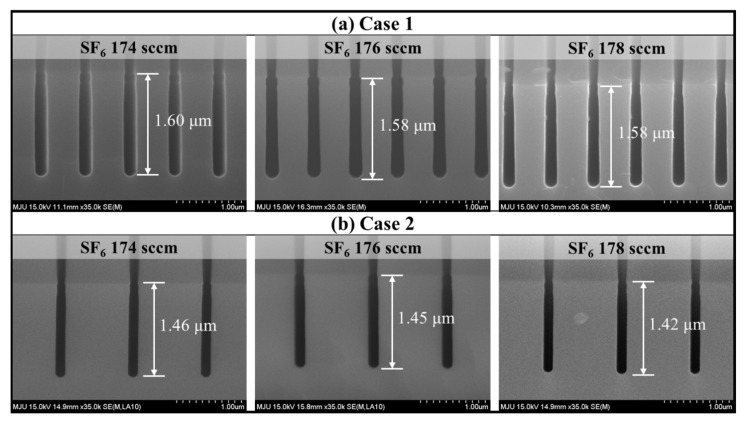
Cross-sectional SEM image about the process results.

As aforementioned, small changes in plasma conduction due to aging of equipment components can affect the process results. In the current study, we selected the MFC for demonstrating the fault detection of the process fault and performed the fault detection of the etching equipment component induced by the degraded MFC. MFC is an important component for controlling the gas injected into the process chamber. We have confirmed that the gas process significantly affects the process result in the previous studies [33,34]. Data from the abnormal process were acquired followed by the experimental scenario. To detect the process faults caused by a minute amount of MFC calibration drift, an abnormal scenario was made by intentionally mis-calibrating the MFC for SF_6_ by 2 sccm from the baseline recipe, as presented in Table 1. Since the MFC of the etching equipment used for data collection has a 1% error and tolerance of the etching limits the gas flow reading to within ±1 sccm, 2 sccm was selected as the minimum unit to assume the minute changes due to aging of the MFC parts. The abnormal process scenario, as shown in Figure 2, confirmed that a 2 sccm change in the MFC set point changed the process result.

**Figure 3:**
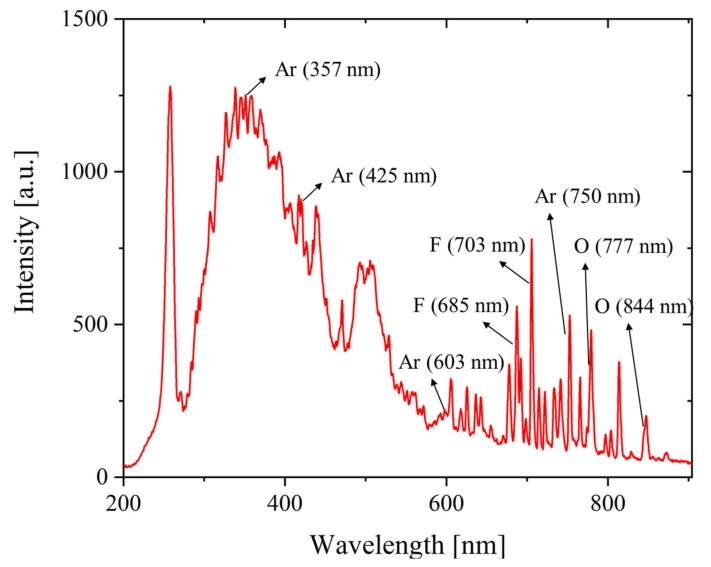
OES full-spectrum and selected wavelengths.

OES is a sensor that measures photon counts in the plasma glow discharge, and the observed plasma emission lights at specific wavelengths explain the chemical species inside the plasma with respect to the wavelengths. The spectroscopic data of plasma glow discharge consist of high-dimensional wavelength variables. It is important to obtain meaningful data from all high-dimensional data measured using OES data. In addition, dimensionality reduction of OES, such as feature selection and extraction, is essential before model learning [35]. Prior to acquiring the abnormal scenario data, we first analyzed the OES data from the normal process recipe and then performed feature selection to store them in the DB with the spectroscopic domain knowledge. The selected OES wavelength features are fluorine (685 and 703 nm), oxygen (777 and 844 nm), and argon (357, 425, 603, and 750 nm), which are eight radical-related peaks generated in the gas plasma process. Figure 3 shows the wavelength axis data of the OES obtained from the normal recipe and indicates the eight pre-selected peaks.

The description of the two OES datasets acquired at different periods is as follows: (1) the first dataset, named Case 1, consists of 120 and 540 normal and abnormal data, respectively, and (2) the second dataset, named Case 2, has 259 and 100 normal and abnormal data. In the data preprocessing, we cleaned the data by removing missing data and outliers from both datasets and performed data scaling using MinMaxScaler. Both datasets have two classes for normal/abnormal processes; the minority classes of Cases 1 and 2 are the normal and abnormal classes, respectively. In the present study, we performed the GAN modeling to generate minority classes for both cases of class imbalance and confirmed the performance variation of the fault detection modeling.

## 3. Data Generation for Class Imbalance

### 3.1. Generative Adversarial Networks

Machine learning can be classified into supervised and unsupervised learning based on the presence or absence of data labels. Unlike supervised learning with labeling, unsupervised learning learns the data distribution when the label is unavailable. The GAN is a deep learning algorithm that learns the distribution of data using unsupervised learning, and it is used to generate artificial data similar to the original data with the same distribution using the existing data.

**Figure 4:**
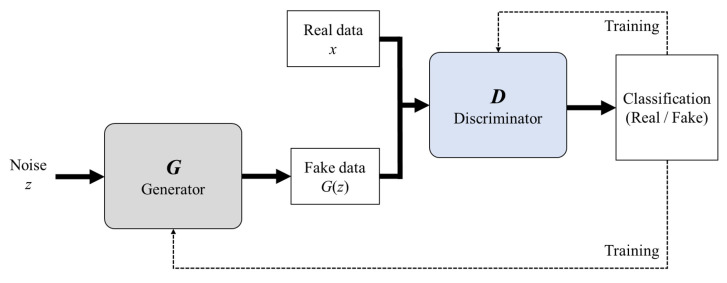
Schematic of the GAN.

As shown in Figure 4, the GAN consists of a generator that generates fake data and a discriminator that separates real from fake data (generated data). Both the generator and discriminator learn to improve their performance through interacting with each other. To train the generator distribution *pg* to fit the data *x*, the noise variables *pz*(*z*) are input into generator *G* to generate the output data *G*(*z*). The discriminator *D* improves the classification performance when real data *x* and generated fake data *G*(*z*) are input. Accordingly, *G* learns to generate data with a distribution similar to *x*. Equation (1) is a value function *V*(*D*, *G*), where *D* is trained to maximize the probability of classifying with the correct label, and *G* is trained to minimize log(1 − *D*(*G*(*z*))) [36].

(1) $$\underset{G}{\min}\;\underset{D}{\max}V\left(D,G\right)=E_{x\sim p_{data}\left(x\right)}\left[\log D\left(x\right)\right]+E_{z\sim p_{z}\left(z\right)}\left[\log\left(1-D\left(G\left(z\right)\right)\right)\right]$$

The GAN aims to help the generator generate realistic data so that the discriminator cannot distinguish the generated data. In this paper, we studied the class imbalance problem of fault detection and classification technology using OES data in the plasma process by applying the GAN to generate minority class data. In each of the two datasets, training data and test data used in fault detection modeling were divided into 8:2, and the minority data in the training data was applied to the GAN.

### 3.2. Modeling of Case 1

Case 1 is a situation where the number of normal cases is smaller than that of abnormal cases, demonstrated by the fault state data of the equipment with an example of the MFC malfunctioning. Here, the normal OES data, which are the minority class of Case 1, were applied to the GAN. The hyperparameters of the final model with the best performance of the GAN model were selected over an extensive model establishment with hyperparameter modifications and model training. The generator that creates fake data by learning the distribution of normal data in Case 1 is composed of an input layer of 10 dimensions, three hidden layers (128-128-256), and an output layer that generates fake data of the eight OES wavelengths. Furthermore, the input noise of the generator is drawn from a uniform distribution with a minimum of zero and a maximum of one. For the activation function, we applied a leaky rectified linear unit, leaky ReLU was applied to each hidden layer [37], and a hyperbolic tangent function was applied to the activation function of the last layer. The discriminator that distinguishes the generated data from the real data in the generator consists of an input layer with eight selected OES wavelengths of fake data, three hidden layers (256-128-128), and an output layer of one node that evaluates whether the data are fake. The leaky ReLU was also applied to the activation function in each hidden layer, and the Sigmoid function was applied to the activation function in the last layer. Weight initialization was performed to help model training by applying the initializer to the generator and discriminator [38].

Binary cross entropy (BCE), as shown in Equation (2), was used as a loss function for model training to classify the normal class (0) and abnormal class (1). Adam was used as the model optimizer, and the learning rate was set to 0.0005 and beta_1 was set to 0.5.

(2) $$BCE=-(y_{i}log\left(h\left(x_{i}\right)\right)+\left(1-y_{i}\right)log\left(1-h\left(x_{i}\right)\right)$$

**Figure 5:**
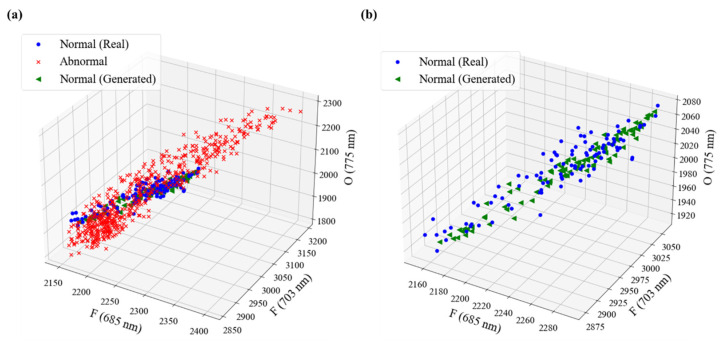
Visualization for generated data using the GAN in the modeling of Case 1; (**a**) real data with generated normal data and (**b**) real normal data and generated normal data.

The batch size of the training data was set to 32 with model epochs set at 41 by checking the loss and metric improvement of the training and test data. The generator and discriminator losses of the final trained model were 0.96 and 0.72, respectively, and the discriminator accuracy was 0.5. The real and normal data generated using the GAN model are visualized, as shown in Figure 5. Among the input features, the OES intensity of three features for fluorine (F*) and oxygen radicals (O*) was expressed in a 3-D graph, and it was confirmed that the distribution of the generated normal data appeared similar to that of the real normal data.

### 3.3. Modeling of Case 2

**Figure 6:**
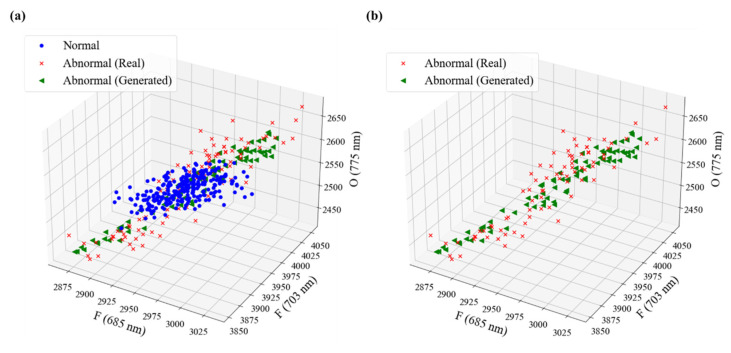
Visualization for the generated data using the GAN in modeling of Case 2; (**a**) real data with generated abnormal data and (**b**) real abnormal data and generated abnormal data.

Case 2 is a dataset in which the abnormal state data of the equipment are smaller than the normal state data, and the minority class is the abnormal class denoted by (1). Similar to the modeling in Case 1, the model with the best performance was selected through hyperparameter tuning. The GAN model structure of Case 2 is the same as Case 1. The model was trained with a batch size of 32 and 180 epochs for training. In the final trained GAN model, the generator and discriminator losses were 0.94 and 0.64, respectively, and the discriminator accuracy was 0.5. The training epochs of the model were terminated with a discriminator accuracy of 0.5, which did not properly classify the fake and real data, as in Case 1. The GAN model of Case 2 generated abnormal data, which is a minority class of Case 2, and the generated abnormal and real data are shown in Figure 6. It was confirmed that the generated abnormal data were formed in a distribution similar to the real abnormal data.

## 4. Fault Detection Modeling

### 4.1. Machine Learning Algorithms

In order to check the effect of the GAN algorithm for solving the class imbalance problem in data modeling for fault detection, six of the most commonly accepted algorithms for the fault detection study were applied, including representative classification algorithms, such as the *k*-nearest neighbor (KNN) and support vector machine (SVM). The KNN algorithm is one of the simplest machine learning algorithms for classifying data based on the similarity of the dataset. The classification of the data class is created by the majority vote of the neighboring data. The KNN has the advantage of being robust to the noise of the training data and is efficient when the training data prevail. However, the number of nearest neighbors (*k*) and distance type must be determined to enhance the modeling accuracy.

The SVM is a representative supervised learning algorithm for finding the optimal hyperplane in the *n*-dimensional space with the largest margin between data classes. Support vectors refer to data points closest to the hyperplane, and the SVM maximizes the margin, which is the distance between the support vector and the hyperplane. The SVM has been used with kernel functions, such as radial basis functions for nonlinear datasets. It sets the regularization *C* and kernel parameters.

Random forest is an ensemble machine learning method using a decision tree algorithm. The ensemble algorithm uses various methods, such as boosting, bagging, and stacking. The random forest is a bagging method that uses the bootstrap sample of the dataset as a parallel structure of a decision tree. Instead of using all features for model training, decision trees are constructed by randomly selecting some features, and a simple majority vote method is used for the model prediction. Its advantages include reducing prediction variability and preventing overfitting. For model learning, hyperparameters related to the decision tree structure, the number of decision trees, and the number of features to be randomly selected should be considered.

The gradient boosting machine (GBM) is another ensemble machine learning method, which is a boosting-based algorithm with a serial structure. The boosting algorithm learns several weak learners sequentially and assigns weights to erroneously predicted data. GBM is trained using gradient descent when updating weights. Among several machine learning algorithms, it is known for its high predictive performance and has been a bedrock for several extended algorithms. In model training, it is necessary to set the learning rate of the gradient descent and the appropriate number of estimators to avoid model overfitting.

The artificial neural network (ANN) is the core technology of deep learning that embodies neurons, which mimic nerve cells. The ANN has a perceptron structure that is applied to the activation function by multiplying input data by weights. The ANN using the backpropagation algorithm in the multilayer perceptron form consists of an input layer, hidden layers, and an output layer. Its advantages include identifying complex relationships between dependent and independent variables and handling noisy data. It is necessary to set the hyperparameters constituting the ANN, including the structure of the hidden layer, type of activation function, and type of optimizer.

### 4.2. Modeling and Evaluation

**Figure 7:**
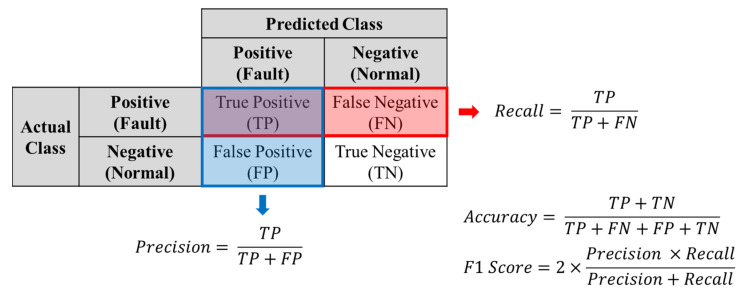
Evaluation metrics for the classification model prediction.

In the silicon trench etching process using SF_6_, O_2_, and Ar plasma, the two datasets were applied for the fault detection modeling of the MFC degradation. Five algorithms were implemented for the fault detection exercises, including 100% oversampling of the minority data generated using the GAN in the training, and their performances were evaluated using the test data consisting of real data. For the four machine learning algorithms except for the ANN, optimal hyperparameters were found using a grid search with cross-validation, and the ANN algorithm was also tuned by hyperparameters with cross-validation to improve model performance. Trained models were evaluated according to accuracy, precision, recall, and F1 score, and the evaluation metrics are shown in Figure 7. In addition, we applied the SMOTE algorithm, which is widely used to alleviate class imbalance, and compared it with the results of the GAN algorithm. The SMOTE algorithm was also applied to the five FDC algorithms after 100% oversampling of the minority class data.

Once the Case 1 dataset was trained with five algorithms, the accuracy performance results of the training and test data are shown in Table 2 (a). For the model training by adding the generated GAN data, the accuracy of the test data increased in all algorithms except the KNN. Moreover, when model training was applied to the generated SMOTE data, the increase in accuracy was insignificant or rather decreased. Even in the KNN model, which showed no improvement through the GAN, the GAN produced a better accuracy than the SMOTE. Judging from the accuracy difference between the training and test data, applying the GAN showed relatively stronger results in overfitting than applying the SMOTE. In classifying the imbalanced data, comparing the accuracy and other performances, such as precision and recall, it is necessary to measure the model performance. Table 2 (b) summarizes the metric results of the test data for all models. Case 1 is a dataset where the minority class belongs to the normal class and is denoted by (0). Machine learning algorithms trained on class imbalanced datasets tend to be biased towards majority classes [39], and the values of the recall metrics are likely to be high in the imbalanced dataset. The recall value was also lowered in training with data generated by the GAN. As the class imbalance was alleviated, the precision value increased in general, and the F1 score also increased. In the KNN, which showed an exception, the GAN still performed better than the SMOTE in the metrics. The ANN that was trained with a precision value higher than the recall value did not decrease the recall value and showed a better performance after applying the GAN. To summarize the results of Case 1, when data of the normal class (0) were artificially augmented with the GAN and applied to the fault detection model, the precision significantly increased, recall decreased, and accuracy and F1 score increased. The normal class (0) data generated using the GAN reduced the learning focused on the abnormal class (1) in the classification model. Therefore, we found that model performance was achieved while the GAN model prevented overfitting due to data generation.

Similarly, five algorithms are applied to the Case 2 dataset, and the accuracy results for the training and test data are shown in Table 3 (a). In models trained with data generated by the GAN, the accuracy of the test data was similar to or increased, whereas in models trained with data generated by the SMOTE, the accuracy of the test data was similar to or decreased. The model trained with the data generated by the SMOTE increased the difference in accuracy between the training data and the test data, resulting in an increase in overfitting. Table 3 (b) shows the model performance of precision, recall, and F1 score before the inclusion of the artificially generated data. Case 2 is a dataset where the minority class belongs to the abnormal class denoted by (1) and the values of recall metrics are likely to be low in the imbalanced dataset. Prior to using the generated minority class data, the precision value of the FDC model was high and the recall value was low. Following the application of the SMOTE and GAN, as the class imbalance problem was alleviated, the precision value decreased and the recall value increased. When learning with data generated by the GAN, the F1 score performance was better than before the generation and the SMOTE. Case 2 is a dataset in which the minority class is an abnormal class similar to the class imbalance problem in real industry. In an actual semiconductor manufacturing environment, the class imbalance in the fault detection problem is more severe than Case 2 shown in this result. Our proposed scenario intended to create a faulty process by modifying intentional twigs of the control knob in SF_6_ gas MFC. However, the number of faulty datasets for high volume manufacturing has a much smaller number of datasets from a specific fault case. We addressed the diagnostic importance of the abnormal state of equipment to reduce manufacturing time and costs leading to wafer failure and equipment maintenance. For fault detection in semiconductor equipment, it is important to show a high recall value. Our results confirmed that the abnormal data generated using the GAN improved the learning of the minority class and improved the classification performance of the model.

### 4.3. Results & Discussion

**Figure 8:**
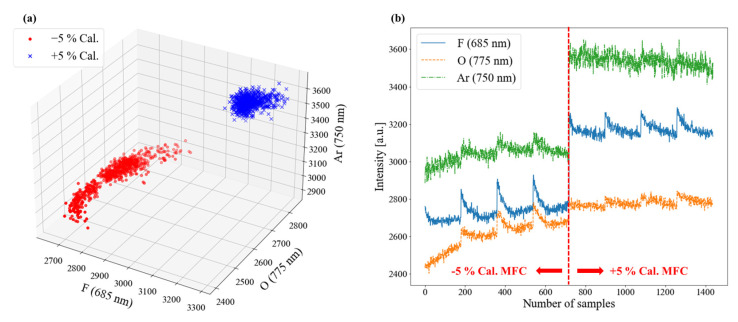
OES data of experiments for the mis-calibrated MFC; (**a**) 3D plot with each mis-calibrated MFC, and (**b**) variation of OES intensity.

**Figure 9:**
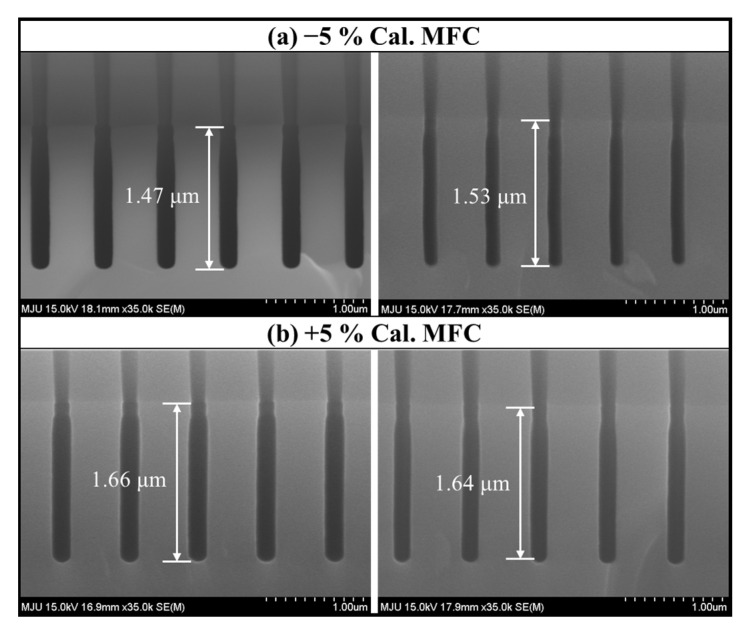
Cross-sectional SEM image of process results with the mis-calibrated MFC; (**a**) results with −5% calibrated MFC and (**b**) results with +5% calibrated MFC.

In this research, we assumed the miscalibration of the MFC due to the age of a semiconductor equipment component and established a model with the acquired data. To increase the validity of our modeling, we conducted additional experiments by obtaining mis-calibrated MFCs by −5% and +5%, respectively. Each mis-calibrated MFC was installed, and the etching process was performed using the normal recipe with the results shown in Figure 8 and Figure 9. As shown in Figure 8a, the distribution of the OES data was different for each MFC, which means that the plasma change was proportional to the degree of miscalibration. In Figure 8b, when changing from −5% to +5% mis-calibrated MFC, the overall intensity of OES, including F (685 nm), O (775 nm), and Ar (750 nm) increases. From Figure 9, it was confirmed that the etch depth increased. Through this additional experiment, we determined that the miscalibration of the MFC affects the process plasma and results, supporting the validity of this study.

Furthermore, we considered the cases of class imbalance in the OES sensor data for Cases 1 and 2, where the minority classes are normal and abnormal, respectively. In each case, the minority data were generated using the GAN and were applied to five machine learning algorithms for fault detection in the semiconductor etching process. In Case 1, the overall precision, accuracy, and F1 score values were increased when the classification model was trained by generating normal class data using the GAN. In Case 2, when abnormal class data were generated, recall, accuracy, and F1 score all increased in some algorithms. In both Case 1 and Case 2, the GAN-generated minority data alleviated the class imbalance problem of the FDC model, and derived a better FDC model performance than the SMOTE-generated data that caused overfitting.

Most of the normal and abnormal data are acquired in the semiconductor industry. However, most are normal data, and there are very few abnormal data with a problem in the equipment, resulting in a class imbalance type, such as in Case 2. As aforementioned, the fault detection of the semiconductor equipment is important for preventing wafer scrap and reducing equipment maintenance time and requires high recall values to predict actual abnormalities. In Case 2, the abnormal data generated using the GAN contributed to an increase in the recall value in some fault detection algorithms. This study is expected to positively affect the data science domain of the semiconductor industry, which suffers from a severe class imbalance. Since the OES analyzes plasma through light in a non-contact manner, it is widely used in semiconductor plasma equipment. However, the problem of class imbalance with respect to OES data has hardly been studied. Therefore, we attempted to mitigate the class imbalance problem of the OES data to establish clearer decision criteria for the fault detection of equipment and stringent control of future semiconductor plasma etching processes. Due to the limitations of the university level, this study targeted 200 nm trench etch and proceeded with a process assuming aging of the component. Currently, sub-50 nm ultra-low silicon trenches are required in the industry [40]. In ultra-low silicon trenches, tighter process conditions will be required than in this study, and if additional plasma analysis data are used based on this study, it is expected to make a great contribution to the actual industry.

## 5. Conclusions

This study proposes a method for solving the class imbalance problem for fault detection using OES data in semiconductor plasma equipment. The GAN was trained to generate considering the distribution of minority class data for fault detection using the acquired OES data. Furthermore, we evaluated the usefulness of the data augmentation of the minority dataset using five fault detection models. Two datasets, each with a normal or abnormal minority class, were applied, and our results showed that the class imbalance problem was alleviated in both cases. The class imbalance problem that prevails in the semiconductor industry occurs when the minority class is abnormal. It is expected that our proposed method significantly contributes to alleviating the data class imbalance problem in fault detection engineering. An artificially generated dataset using the GAN helps to balance the dataset size of the two or more classes in a classification problem, and machine learning models learn better with the prevention of overfitting during the machine learning step. Furthermore, we demonstrated the degradation of one equipment part by intentionally controlling the set value of the MFC for SF_6_. Through a series of silicon trench etching processes with SF_6_/O_2_/Ar plasma, employing 300 mm high volume manufacturing etching equipment, we collected OES data for the monitoring of plasma glow discharge. Considering the university research environment, only one of the various equipment components, the MFC, was investigated; nonetheless, the detection of the plasma disturbance from other degradation parts still conceptually holds. Based on this study, it is required to compare various process parameters for the actual aging data of plasma equipment components in a nanoscale process for future application in the industry.

## Figures and Tables

**Table 1:** Normal/abnormal scenario process recipe.

Experiment Recipe		Case 1	Case 2
Power [Watt]	Source	800	800
	Bias	50	50
Pressure [mTorr]		20	20
Gas Flow [sccm]	SF_6_	158–194 (Normal: 176)	170–182 (Normal: 176)
	O_2_	124	124
	Ar	10	10
Chuck Temperature [°C]		25	25
Time [second]		180	180

**Table 2:** Fault detection model performance with Case 1; (**a**) model accuracy with training data and test data and (**b**) model performance with test data.

**(a) Case 1 Model Accuracy [%]**									
**Algorithm**	**Before Generation**			**After SMOTE**			**After GAN**		
	**Train** **Data**		**Test** **Data**	**Train** **Data**		**Test** **Data**	**Train** **Data**		**Test** **Data**
Random Forest	97.0		90.2	97.5		90.9	98.1		91.7
Gradient Boosting Machine	97.2		91.7	98.9		90.2	97.0		92.4
Support Vector Machine	94.5		91.7	94.1		89.4	92.8		92.4
K-Nearest Neighbors	95.1		95.5	93.8		93.2	93.9		94.7
Artificial Neural Network	96.8		93.9	97.7		93.9	97.7		94.7
**(b) Case 1 Model Performance [%]—Test Data**									
**Algorithm**	**Before Generation**			**After SMOTE**			**After GAN**		
	**Precision**	**Recall**	**F1 Score**	**Precision**	**Recall**	**F1 Score**	**Precision**	**Recall**	**F1 Score**
Random Forest	92.8	95.4	94.1	95.3	93.5	94.4	95.3	94.4	94.9
Gradient Boosting Machine	93.7	96.3	95.0	94.4	93.5	94.0	93.8	97.2	95.5
Support Vector Machine	94.5	95.4	94.9	96.1	90.7	93.3	97.1	93.5	95.3
K-Nearest Neighbors	97.2	97.2	97.2	99.0	92.6	95.7	99.0	94.4	96.7
Artificial Neural Network	97.2	95.4	96.3	95.5	97.2	96.3	97.2	96.3	96.7

**Table 3:** Fault detection model performance with Case 2; (**a**) model accuracy with training data and test data and (**b**) model performance with test data.

**(a) Case 2 Model Accuracy [%]**									
**Algorithm**	**Before Generation**			**After SMOTE**			**After GAN**		
	**Train** **Data**		**Test** **Data**	**Train** **Data**		**Test** **Data**	**Train** **Data**		**Test** **Data**
Random Forest	94.4		88.9	96.9		88.9	94.8		90.3
Gradient Boosting Machine	96.5		90.3	96.9		90.3	92.3		91.7
Support Vector Machine	94.8		91.7	95.1		88.9	93.7		91.7
K-Nearest Neighbors	92.3		91.7	94.4		90.3	93.0		91.7
Artificial Neural Network	94.4		90.3	95.5		90.3	93.4		91.7
**(b) Case 2 Model Performance [%]—Test Data**									
**Algorithm**	**Before Generation**			**After SMOTE**			**After GAN**		
	**Precision**	**Recall**	**F1 Score**	**Precision**	**Recall**	**F1 Score**	**Precision**	**Recall**	**F1 Score**
Random Forest	92.9	65.0	76.5	87.5	70.0	77.8	93.3	70.0	80.0
Gradient Boosting Machine	93.3	70.0	80.0	84.2	80.0	82.1	93.8	75.0	83.3
Support Vector Machine	100.0	70.0	82.4	87.5	70.0	77.8	93.8	75.0	83.3
K-Nearest Neighbors	100.0	70.0	82.4	88.2	75.0	81.1	93.8	75.0	83.3
Artificial Neural Network	100.0	65.0	78.8	100.0	65.0	78.8	93.8	75.0	83.3

## Data Availability

The data presented in this study are available upon request from the corresponding author. The data are not publicly available due to the restriction of the equipment supplier.
